# Menopausal Symptoms in Underserved and Homeless Women Living in Extreme Temperatures in the Southwest

**DOI:** 10.1089/whr.2020.0083

**Published:** 2021-03-16

**Authors:** Mahnoor Mukarram, Veena Rao, Maheeyah Mukarram, David M. Hondula, Matthew R. Buras, Juliana M. Kling

**Affiliations:** ^1^ Barrett, The Honors College at Arizona State University, Tempe, Arizona, USA.; ^2^ School of Geographical Sciences and Urban Planning Arizona State University, Tempe, Arizona, USA.; ^3^ Department of Health Sciences Research, Mayo Clinic Arizona, Scottsdale, Arizona, USA.; ^4^ Division of Women's Health Internal Medicine, Mayo Clinic Arizona, Scottsdale, Arizona, USA.

**Keywords:** climate, heat, homeless, low-income, menopause, summer, uninsured, vasomotor symptoms

## Abstract

**Background:**

Little is known about menopausal symptoms in underserved women.

**Aim:**

To better understand self-reported menopausal symptoms in underserved and homeless women living in extreme heat during different seasons.

**Methods:**

A cross-sectional study, including the Greene Climacteric Scale (GCS), climate-related questions, and demographics was administered June to August of 2017 and December to February 2018 to women 40–65 years of age.

**Results:**

In 104 predominantly Hispanic (56%), uninsured (53%), menopausal (56%), and mid-aged (50 ± 9.5) women, 57% reported any bother, while 20% of these women reported “quite a bit” or “extreme” bother from hot flushes. The total GCS score was *n* = 104: Mean (SD) 19.8 (15.3); out of 63 indicating significant symptoms, the psychological and somatic clusters were highest. Women did not think *temperature* outside influenced their menopausal symptoms at either time point (69% in winter vs. 57% in summer, *p* = 0.23). In multivariable analyses after adjusting for race, body mass index, and living situation neither season nor temperature was associated with self-reported hot flush bother. While one-third of women reported becoming ill from the heat, 90% of women reported not seeking care from a doctor for their illness.

**Conclusion:**

Menopausal, underserved, homeless women living in Arizona reported few vasomotor symptoms regardless of season, and endorsed psychological and somatic complaints. Socioeconomic factors may influence types of bothersome menopausal symptoms in this population of women.

## Introduction

Menopause can be associated with emotional, physical, and cognitive changes, including hot flushes and night sweats, also referred to as vasomotor symptoms (VMS).^1,2^ Oftentimes, bother from VMS is used to characterize degree of symptoms as it is the most common menopausal symptom.^3^ Women from different geographies and from ethnically diverse populations show different VMS frequency and characteristics, which may be partially explained by regional and geographical differences.^4^ For example, prevalence of VMS in North America has been reported to differ by ethnicity with the lowest for women of Japanese ethnicity (18%), and higher numbers for Caucasian (31%), Hispanic (35%), and African American women (46%).^5^ In Europe, the prevalence of hot flushes has been reported at 73%, whereas in Latin America the numbers are less clear and range from no VMS (in Mayan women in Mexico) to 67% of Mayan women in Guatemala reporting hot flushes.^5^ Season may also influence symptoms by contributing to increases in VMS and sleep troubles in summer months due to heat compared to winter months.^6^ It is hypothesized that women develop climate-specific thermoneutral zones leading to population-specific frequencies of hot flushes.^7^ These climate-specific factors may influence the timing of and characteristics related to menopause.^7–10^

In addition to climate, intersectional demographic factors such as ethnicity may also play a difference in VMS frequency and characteristics. The Study of Women's Health Across the Nation (SWAN) evaluated menopausal symptoms in multiethnic women and found that Hispanic women reported more VMS than non-Hispanic Caucasians, noting that Central American women were at greatest risk for VMS.^11^ Another study also found ethnic differences in VMS among non-Hispanic whites, Hispanic, non-Hispanic African Americans, and non-Hispanic Asians.^12^ Women of Brazilian descent were more likely to report VMS with a prevalence more similar to western women, whereas few Asian women reported menopausal symptoms and Mayan women did not report VMS at all.^12^ In peri- and postmenopausal women in rural and urban areas of three States of Mexico identified that the most frequent menopausal symptoms reported were headache, anxiety, and muscular pains, with VMS reported by less than half interviewed.^13^ Another study found that while the majority of Mayan women reported hot flushes, they do not differentiate or separate expression of hot flashes from their lived experience of the hot climate, possibly because hot flushes are neutralized by the hot and humid environment in everyday life.^14^

Beyond ethnicity, only a few studies have evaluated other socioeconomic factors that may impact menopausal symptoms in women, such as lack of access to health insurance or homelessness. One evaluating socioeconomic status (SES) conducted at an inner-city menopause clinic found that Hispanic postmenopausal women of low SES had a high prevalence of female sexual dysfunction (75.6%), a symptom oftentimes related to the genitourinary syndrome of menopause.^15^ Furthermore, a study of 351 postmenopausal women in rural North India demonstrated that many variables such as low SES, as well as low educational attainment, advancing age, later age at menarche, and higher body mass index (BMI), were all associated with more severe menopausal symptoms.^16^ No prior studies have evaluated menopausal symptoms in underserved women living in an area of extreme temperature in the Southwest United States. Furthermore, it is unclear if other socioeconomic factors such as homelessness impacts menopausal symptoms.

Our study's aim was to understand self-reported menopausal symptoms in underserved women in a region that experiences extreme heat. For purposes of this study, underserved women include those who are medically underserved as one or a combination of being uninsured, attending free, and low-income clinics, or currently homeless. In the United States, heat is a leading weather-related cause of human mortality and morbidity.^17^ During the summer months, Arizona may experience heat over 100° Fahrenheit, with the maximum heat being 122° Fahrenheit.^18^ The city of Phoenix specifically has been documented to have had the most extreme case of heat exposure intensity, partially driven by region and climate.^17^ Individuals of lower SES experience higher outdoor heat exposure and as a result face increased risk of negative heat-related health outcomes.^17^ Homeless individuals—women in particular—are at increased risk of heat-related and morbidity and mortality due to greater time outdoors in the heat.^17,19^ Harsh outdoor living conditions and associated hazards to their health and well-being result in such as dehydration, thermal discomfort, fatigue and exhaustion, cardiovascular and respiratory distress, heat stroke, and other heat-related illnesses.^17,20^ Their vulnerability to environmental risks because of exposure to poor living conditions in turn can negatively affect their health.^21^

Evaluating responses to menopausal symptom questions during months of extreme heat, and comparing that to responses during cooler months may help provide insight into how temperature and season may impact menopausal symptoms in this vulnerable population. The season refers to winter or summer, whereas the temperature refers to heat or lack of heat outside. In this study, women in free, low-income, and homeless clinics in downtown Phoenix, Arizona, were surveyed during both winter and summer months using a validated menopause questionnaire.

## Methods

A descriptive cross-sectional study was performed using a self-administered written survey during the summer months of June to August of 2017 and the winter months of December to February 2018. The survey was anonymous and no personal identifiers were collected. The Arizona State University Institutional Review Board (IRB) deemed the project exempt before the administration of surveys (STUDY00005355). Potential harms included personal discomfort in answering sensitive questions relating to menopause symptoms, therefore participants were interviewed in privacy, including in examination rooms. Participant selection and recruitment criteria were based on age. Inclusion criteria included women aged 40–65 years either attending the clinic or accompanying patients who were attending the clinic. Exclusion criteria included women younger than 40 years or older than 65 years of age and men. The reason for the visit was not obtained for each participant and was not dependent on them being there to discuss menopausal symptoms. Prior permission from management at each institution was received as well as informed consent from participants. Participants were clearly informed that their participation was voluntary and that there was no obligation to complete the survey. No incentive was provided for participation and it took ∼15 minutes to complete the survey. A Spanish speaker from Mexico translated surveys into Spanish and a research coordinator facilitated questionnaire completion. Refusals to participate were not noted and all completed anonymous surveys were recorded in one database for analysis. English and Spanish versions of the questionnaire were available upon patient request.

Surveys were administered on-site by the study coordinator and interpreters to clinic patients at St. Vincent de Paul medical clinic and Parson's Family Health Center at Circle the City. The St. Vincent de Paul clinic is available to those without health insurance and serves predominantly a Spanish-speaking and undocumented population. The Parson's Family Health Center serves people who are homeless, and many have health insurance through Medicaid. The Area Deprivation Index (ADI) shows that the neighborhood immediately surrounding the St. Vincent de Paul Clinic falls into the most disadvantaged (10th decile) in Arizona, and the 97th percentile nationally. The neighborhood surrounding the Parson's Clinic has an ADI score in the 7th decile in Arizona, and nationally is the 72nd percentile. To quantify neighborhood disadvantage, 17 parameters are used in the ADI, including but not limited to education, poverty measures, employment, and housing quality.^18,19,22^

Demographic and personal health history questions were included. Menopause stage was determined by asking participants if they had a menstrual cycle in the last 12 months (yes or no) or in the last 3 months (yes or no), as well as the date of their last menstrual cycle. Participants were also asked if they are taking birth control pills, using an intrauterine device, on hormone therapy (HT), or taking any medication for menopause symptoms. They were also asked if they had surgery to remove their uterus or surgery to remove both of their ovaries.

The survey included the Greene Climacteric Scale (GCS), a validated menopause survey questionnaire, as well as climate-related questions and demographics. The GCS questionnaire is validated and has been used in both English and Spanish speaking countries to evaluate climacteric-related factors in postmenopausal women.^2,7,20,23–25^ GCS total and cluster scores (psychological, somatic, vasomotor, and sexual interest) were calculated. Higher scores are indicative of more symptoms. Each symptom is rated according to current severity using a four-point rating scale: not-at-all (0); a little (1); quite a bit (2); extremely (3). The total GCS score per subject is the sum of all 21 scores.

Climate questions, which refer to all temperature-related questions, were developed based on a literature review and expert input, then revised from staff suggestions to assure clarity and readability. In an open-response and multiple-choice format, participants were asked about the length of time they lived in Arizona, the zip code they resided in, and if they ever experienced medical illness due to heat. If they responded “yes,” they were asked about their symptoms, if and how they self-treated at home, and whether they sought treatment from a physician. The climate portion of the survey also asked participants their opinion on heat risk and whether they believed that the current season and temperature outside influenced their menopausal symptoms.

Sample size was estimated using the Cohen h effect size with the statistical test being a two-tailed 2 independent samples proportions test to detect a statistically significant difference between GCS scores in winter versus summer. It was determined that 63 patients are required to detect an effect of size 0.5 or larger with 80% power, assuming a significant level of 0.05.

Numeric variables were summarized by mean and standard deviation while categorical variables were quantified by frequency and percent. For continuous variables, the equal variance *t*-test was used to compare the mean between two groups and the one-way ANOVA was used in the case of more than two groups. The distribution of the responses of categorical variables was compared between groups using the chi-squared test. Multivariable linear models adjusting for BMI, living situation, and race were used to test the association between temperature and season with GCS scores and degree of bother experienced by hot flushes.

Subanalysis to evaluate responses by living situation (homeless vs. not) and insurance status (yes vs. no) were also conducted to further investigate factors that may be associated with GCS outcomes. All hypotheses tested were two-sided with *p* < 0.05 considered statistically significant. There was no adjustment for multiple testing. Analyses were performed in SAS v9.4 (SAS Institute, Inc., Cary, NC).

## Results

A total of 104 predominantly Hispanic, uninsured, menopausal, middle-aged women were surveyed. In the summer, 54 surveys were collected, and 50 surveys were collected in the winter, approximately half at each site (Table 1). At the time of the survey, 22% of participants were homeless or living in a shelter. Most women were not taking menopausal HT (95%) or birth control pills (92%). Hispanics were more likely to be uninsured than other ethnicities (91% uninsured, *p* < 0.001).

**Table 1.** Participant Characteristics (*n* = 104) by Age, Race, Body Mass Index, Living Situation, Insurance, Hysterectomy Status, Hormone Replacement Therapy, Birth Control, and Greene Climacteric Scale

	Summer (*N* = 54)	Winter (*N* = 50)	*p*
	*n* (%)	*n* (%)	
Age			
Mean (SD)	51.7	50.6	<0.31
Range	40–64	40–65	
Race			
White	13 (24.1)	16 (32.7)	<0.00^*^
African American	7 (7.4)	5 (10.2)	
Asian	0 (0.00)	4 (8.2)	
Hispanic	**37 (68.5)**	**19 (38.8)**	
Body mass index			
Mean (SD)	32.5 (8.7)	28.5 (8.0)	0.02^*^
Range	17.6–53.4	15.1–45.9	
What is your current living situation?			
House	**25 (48.1)**	**18 (40.0)**	0.00^*^
Apartment	11 (21.2)	10 (22.2)	
Homeless	15 (28.8)	6 (13.3)	
Other (shelter, roof-top)	1 (1.9)	11 (24.4)	
Do you currently have health insurance?			
No	**35 (64.8)**	**18 (39.1)**	0.01^*^
Yes (type below)	19 (35.2)	28 (60.9)	
Have you had a menstrual period in the last 12 months?			
Yes	21 (41.2)	21 (42.9)	0.86
No	**30 (58.8)**	**28 (57.1)**	
Have you had surgery to remove your uterus (hysterectomy)?			
Yes	5 (9.6)	7 (14.3)	0.46
No	**47 (90.4)**	**42 (85.7)**	
Are you currently taking hormone therapy for menopause?			
Yes	2 (3.8)	1 (2.0)	0.60
No	**51 (96.2)**	**48 (98.0)**	
Are you currently taking birth control pills?			
Yes	2 (4.1)	0 (0.0)	0.15
No	**47 (95.9)**	**49 (100)**	
Extent to which you are bothered at the moment by these symptoms: (Greene Climacteric Scale)			
Hot flashes			
Not at all	**21 (42.9)**	**24 (57.1)**	0.58
A little	15 (30.6)	10 (23.8)	
Quite a bit	9 (18.4)	6 (14.3)	
Extremely	4 (8.2)	2 (4.8)	
Sweating at night			
Not at all	**18 (38.3)**	**21 (48.8)**	0.72
A little	13 (27.7)	11 (25.6)	
Quite a bit	10 (21.3)	6 (14.0)	
Extremely	6 (12.8)	5 (11.6)	
Greene Climacteric Scale score, mean (SD)			
Total	19.0 (15.2)	21.0 (15.6)	0.54
Psychological cluster	9.7 (8.5)	11.5 (9.1)	0.41
Anxiety subcluster	4.9 (4.3)	5.3 (4.5)	0.76
Depression subcluster	4.8 (4.6)	6.2 (5.1)	0.21
Somatic cluster	6.4 (6.1)	7.3 (6.2)	0.56
Vasomotor cluster	2.1 (1.9)	1.5 (2.0)	0.11
Sexual interest cluster	0.8 (1.0)	0.8 (1.1)	0.83

Bolded answers indicate the majority answer.

^*^ Significant *p*-value.

### Menopausal symptoms during both winter and summer (GCS)

Twenty percent of women reported, “quite a bit” or “extreme” bother from hot flushes. The total GCS score was *n* = 104: Mean (SD) 19.8 (15.3), consistent with a medium level of symptom bother, with the highest scores in the psychological, somatic, depression, and anxiety clusters (Table 1). Table 2 displays patient demographics by self-reported hot flush bother.

**Table 2.** Participant Demographics and Responses (*n* = 104) by Self-Reported Hot Flush Frequency

	Not at all (*N* = 45)	A little (*N* = 25)	Quite a bit (*N* = 15)	Extremely (*N* = 6)	Total (*N* = 91)	*p*
Season, *n* (%)						
Cold	24 (53.3%)	10 (40.0%)	6 (40.0%)	2 (33.3%)	42 (46.2%)	0.585^a^
Hot	21 (46.7%)	15 (60.0%)	9 (60.0%)	4 (66.7%)	49 (53.8%)	
Homeless, *n* (%)						
No	34 (81.0%)	20 (90.9%)	9 (60.0%)	4 (66.7%)	67 (78.8%)	0.124^a^
Yes	8 (19.0%)	2 (9.1%)	6 (40.0%)	2 (33.3%)	18 (21.2%)	
Missing	3	3	0	0	6	
Living, *n* (%)						
House	19 (45.2%)	13 (59.1%)	4 (26.7%)	1 (16.7%)	37 (43.5%)	0.483^a^
Apartment	9 (21.4%)	5 (22.7%)	3 (20.0%)	2 (33.3%)	19 (22.4%)	
Other (shelter, roof-top)	6 (14.3%)	2 (9.1%)	2 (13.3%)	1 (16.7%)	11 (12.9%)	
Homeless	8 (19.0%)	2 (9.1%)	6 (40.0%)	2 (33.3%)	18 (21.2%)	
Missing	3	3	0	0	6	
Race, *n* (%)						
White	13 (29.5%)	4 (16.0%)	4 (26.7%)	4 (66.7%)	25 (27.8%)	0.265^a^
African American	2 (4.5%)	2 (8.0%)	2 (13.3%)	1 (16.7%)	7 (7.8%)	
Asian	2 (4.5%)	0 (0.0%)	2 (13.3%)	0 (0.0%)	4 (4.4%)	
Hispanic	24 (54.5%)	18 (72.0%)	6 (40.0%)	1 (16.7%)	49 (54.4%)	
Other	3 (6.8%)	1 (4.0%)	1 (6.7%)	0 (0.0%)	5 (5.6%)	
Missing	1	0	0	0	1	
Insurance, *n* (%)						
No	21 (50.0%)	19 (76.0%)	6 (42.9%)	2 (33.3%)	48 (55.2%)	0.077^a^
Yes	21 (50.0%)	6 (24.0%)	8 (57.1%)	4 (66.7%)	39 (44.8%)	
Missing	3	0	1	0	4	
Heat, *n* (%)						
0	0 (0.0%)	1 (50.0%)	0 (0.0%)	0 (0.0%)	1 (5.9%)	0.131^a^
Dizziness	1 (12.5%)	0 (0.0%)	2 (50.0%)	0 (0.0%)	3 (17.6%)	
Weakness/exhaustion	4 (50.0%)	0 (0.0%)	0 (0.0%)	2 (66.7%)	6 (35.3%)	
Nauseous/upset Stomach	1 (12.5%)	0 (0.0%)	1 (25.0%)	1 (33.3%)	3 (17.6%)	
Excessive sweating	0 (0.0%)	1 (50.0%)	0 (0.0%)	0 (0.0%)	1 (5.9%)	
Hallucination	1 (12.5%)	0 (0.0%)	0 (0.0%)	0 (0.0%)	1 (5.9%)	
12	0 (0.0%)	0 (0.0%)	1 (25.0%)	0 (0.0%)	1 (5.9%)	
43163	1 (12.5%)	0 (0.0%)	0 (0.0%)	0 (0.0%)	1 (5.9%)	
Missing	37	23	11	3	74	
Vasomotor symptom cluster score by GCS						
*N*	38	18	14	6	76	<0.001^b^
Mean (SD)	0.2 (0.54)	2.2 (0.88)	3.9 (0.77)	6.0 (0.0)	1.8 (1.98)	
Median	0.0	2.0	4.0	6.0	1.0	
Range	0.0, 2.0	1.0, 4.0	2.0, 5.0	6.0, 6.0	0.0, 6.0	

^a^ Chi-square *p*-value; ^b^Kruskal–Wallis *p*-value; cluster_vas_score used as menopausal status.

GCS, Greene Climacteric Scale.

When comparing the groups between summer and winter, a few statistically significant differences were demonstrated between the women (Table 1). Women surveyed in the summer were of a higher BMI, more likely to be Hispanic, and differed by housing situation. Reported GCS symptoms were not statistically significant between the summer and winter months.

### Climate questions

**FIG. 1.**
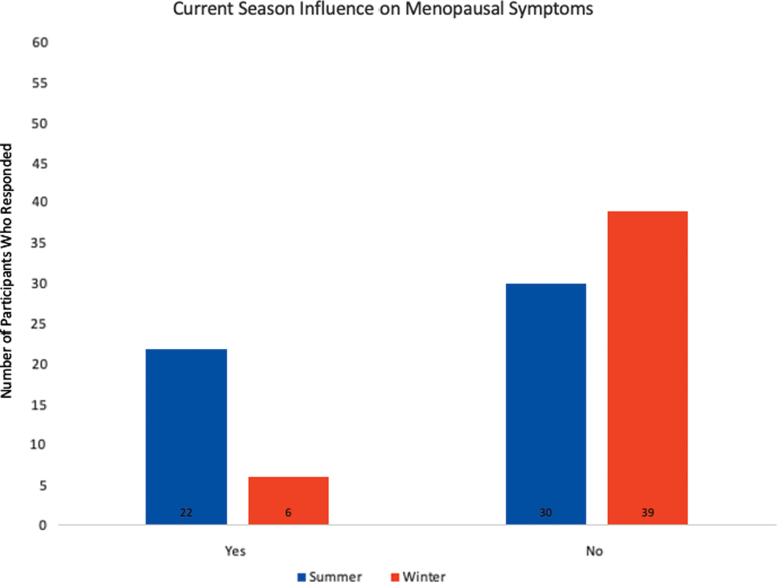
Participant responses for influence of season on menopausal symptoms.

**FIG. 2.**
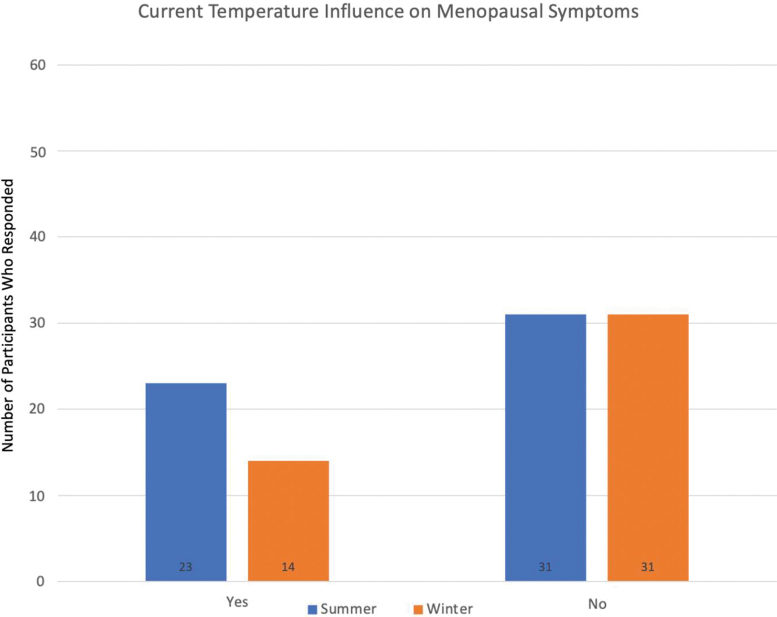
Participant responses for influence of temperature outside on menopausal symptoms.

One-third of women reported ever becoming ill from the heat, but most did not seek treatment for their symptoms related to heat (Table 3). More women thought *season* influenced menopausal symptoms during summer than winter (42% vs. 13%, *p* = 0.23) (Fig. 1). However, a majority of women did not think outdoor temperature influenced their menopausal symptoms and that percentage did not statistically differ by season (69% in winter vs. 57% in summer, *p* = 0.23) (Fig. 2). No statistically significant differences were seen for reported VMS between winter and summer. In multivariable analyses after adjusting for race, BMI and living situation neither season nor temperature was associated with self-reported hot flush bother or any of the GCS domains.

**Table 3.** Climate and Health-Related Questions in Relationship to Heat, Season, and Temperature (*n* = 104)

	Summer, *n* (%)	Winter, *n* (%)	*p*
Have you been medically ill due to heat?			
Yes	15 (27.8)	16 (32.0)	0.63
No	**39 (72.2)**	**34 (68.0)**	
What symptoms did you experience when you were ill due to heat?			
Dizziness	4 (30.8)	0 (0.0)	0.12
Weakness/Exhaustion	4 (30.8)	4 (40.0)	
Nauseous/Upset Stomach	**3 (23.1)**	**0**	
Hallucinations	1 (7.7)	0	
Missing	41	45	
Did you seek treatment from a physician due to heat symptoms?			
Yes	5 (9.6)	4 (8.7)	0.87
No	**47 (90.4)**	**47 (91.3)**	
Did you seek treatment at the Emergency Room (ER) due to heat symptoms?			
Yes	4 (7.7)	9 (19.1)	0.09
No	**48 (92.3)**	**38 (80.9)**	
Do you think the current season influences your menopausal symptoms?			
Yes	22 (42.3)	6 (13.3)	0.23
No	**30 (57.7)**	**39 (86.7)**	
Do you think heat is a risk to your health?			
Yes	**33 (64.7)**	**27 (55.1)**	0.32
No	18 (35.3)	22 (44.9)	
Do you think the temperature outside influences your menopausal symptoms?			
Yes	23 (42.6)	14 (31.1)	0.23
No	**31 (57.4)**	**31 (68.9)**	

Bolded answers indicate the majority answer.

^*^ significant p-value.

### Subanalysis

In a subanalysis that evaluated responses by living situation (homeless vs. not), and insurance status (yes vs. no), statistically significant differences were seen. On the GCS, uninsured women reported fewer symptoms overall compared to insured women (Insurance - Mean (SD): 27.2 (16.0); No Insurance - Mean (SD): 15.6 (13.1); *p* = 0.003). Homeless women reported more symptoms on the GCS compared to non-homeless women (Homeless - Mean (SD): 28.7 (17.8); Not Homeless: Mean (SD): 18.0 (14.0); *p* = 0.05).

## Discussion

In a sample of predominately Hispanic women who are homeless and/or uninsured living in a region that experiences extreme heat, most did not report bothersome hot flushes or night sweats and were not on menopausal HT. The women did not think that the temperature impacted their symptoms during both warm and cooler seasons, which was also supported by multivariable analysis results. Overall, symptom scores were influenced more by factors such as depression, anxiety, and somatic symptoms, which may be related to a compromised quality of life or life stressors. Relationships between increased menopausal symptoms and poorer quality of life have been described, while mindfulness and stress reduction has been shown to be associated with fewer menopausal symptoms.^26^ A study evaluating menopausal symptoms in Ecuadorian women in a low SES not on menopausal HT found participants frequently reported hot flushes, but, similar to our study, reported many somatic symptoms, including headaches, difficulty concentrating, and feelings of unhappiness and distress (*n* = 385: 87%, 83.9%, 82%, and 82%, respectively).^9^ It is plausible that geographic location or even ethnicity may influence VMS perception differently, while socioeconomic factors similarly influence women of lower SES.^4^

During the summer, underserved and homeless women in Arizona were more likely to report that the current season influenced their menopausal symptoms than in winter, but did not think the temperature outside influenced their menopausal symptoms when asked either in summer or in winter. So, although the women thought that the current summer season impacted their menopausal symptoms, they did not think the hot temperature influenced the symptoms, which were interesting findings. While both cold temperatures and heat waves have had negative effects on mortality, moderately high temperatures contribute to the majority of the total health burden caused by temperature.^27^ One may presume that due to the very high temperatures in Arizona during summer that women may notice an impact on symptoms. It has been shown that the influence of unexpected temperature changes may be more impactful, however, than the absolute temperature itself on health,^27^ which has been demonstrated in studies on the impact of heat exposure in vulnerable populations.^21,28–30^ Extreme heat has become a leading cause of death due to weather, as well as lack of personal awareness of the physiologic symptoms of heat intolerance. A study of homeless individuals showed that 55% of those surveyed could not identify symptoms associated with heat illness despite being concerned about their health during times of heat.^28^ Thus, women may get used to or acclimate to the high temperatures during summer and thus do not notice a relationship with symptoms, and indeed changes in temperature may be a factor influencing perception of bother, consistent with prior research that has evaluated relationships of hot flushes and climate.^1^

Despite most women not reporting bothersome hot flushes or night sweats, scores for other symptoms, including depression and somatic symptoms, were relatively high. Furthermore, homeless women reported more symptoms by GCS compared to nonhomeless women. These findings may be related to unique socioeconomic factors faced by the population of underserved and homeless women who were surveyed. Studies have shown that underserved women face a myriad of different health challenges that are the result of factors, including low SES, governmental policies, mental illness, and low education levels.^31–33^ These factors not only contribute to homelessness and being underserved, but may also impact a woman's health.^31,33^ Homeless women are more likely to have lower levels of education, suffer from poor mental health, and be undernourished.^31^ Mental illness is a major factor that can contribute to homelessness and can also significantly impact health.^32^ In western countries, the prevalence of mental illness among the homeless population, including psychotic illnesses and personality disorders, is higher compared to the age-matched general population in those countries.^34^ Homeless mentally-ill women are one of the most deprived populations. A report by Human Rights Watch recognized stigma, discrimination, and a disparity in governmental community-based services and awareness of services as factors that lead to institutionalization.^35^ Our findings point to the need for access to comprehensive behavioral and whole person care models, which ultimately may most improve the lives of midlife homeless and underserved women, including for midlife women in the Southwest.

### Strengths and limitations

The strengths of this study include that it focused on a group of women who have not been evaluated robustly, specifically underserved and homeless women. There is a dearth of information on this patient population, thus this research adds novel insight to the field. In addition, surveys were available in both English and Spanish to cater to the needs of participants.

One of the limitations of our study is a small sample size, which limits the generalizability of the findings. The differences in results between summer and winter populations could be explained randomly as a result of the small sample size. It can be argued that similar result variance could have been found in two summer groups. Moreover, no physiological measurements of participants were made; and observational, self-reported data were used, so the results are susceptible to recall bias. All health information was also based on self-report, so it cannot be independently confirmed. Finally, confounding variables were limited to what was included in the questionnaire.

Recommended steps for future research include obtaining a larger sample size of geographically diverse participants. In addition, it may be beneficial to conduct the study in a longitudinal manner following participants who were initially interviewed in the summer months and reinterviewing them in the winter months.

## Conclusion

Little is known about self-reported menopausal symptoms in homeless and underserved women, especially in the context of the influence of extreme heat. In menopausal, underserved, and homeless women living in Arizona, not many menopausal women reported VMS even during the summer season, which experiences extreme heat. Socioeconomic factors are likely influencing the type of menopausal symptoms that cause bother in this population of women and comprehensive behavioral and whole person care models may most improve the lives of midlife homeless and underserved women.

## Abbreviations Used

ADI: Area Deprivation Index
BMI: body mass index
GCS: Greene Climacteric Scale
HT: hormone therapy
SES: socioeconomic status
SWAN: Study of Women's Health Across the Nation
VMS: vasomotor symptoms

## References

[1] Sievert LL, Flanagan EK. Geographical distribution of hot flash frequencies: Considering climatic influences. Am J Phys Anthropol 2005;128:437–443. 15838836 10.1002/ajpa.20293

[2] Greene JG. Constructing a standard climacteric scale. Maturitas 1998;29:25–312. 9643514 10.1016/s0378-5122(98)00025-5

[3] Sherman S. Defining menopausal transition. Am Journal Med 2005;118:3–7. 10.1016/j.amjmed.2005.11.002 16414321

[4] Gold EB, Bromberger J, Crawford S, et al. Factors associated with age at natural menopause in a multiethnic sample of midlife women. Am J Epidemiol 2001;153:865–874. 11323317 10.1093/aje/153.9.865

[5] Freeman EW, Sherif K. Prevalence of hot flushes and night sweats around the world: A systematic review. Climacteric 2007;10:197–214. 17487647 10.1080/13697130601181486

[6] Harlow SD, Elliott MR, Bondarenko I, Thurston RC, Jackson EA. Monthly variation of hot flashes, night sweats, and trouble sleeping effect of season and proximity to the final menstrual period (FMP) in the SWAN Menstrual Calendar substudy. Menopause 2020;27:5–13. 31567864 10.1097/GME.0000000000001420 PMC6934911

[7] Harlow SD, Gass M, Hall JE, et al. Executive summary of the Stages of Reproductive Aging Workshop +10: Addressing the unfinished agenda of staging reproductive aging. Menopause 2012;19:387–395. 22343510 10.1097/gme.0b013e31824d8f40 PMC3340903

[8] Avis NE, Zhao X, Johannes CB, et al. Correlates of sexual function among multi-ethnic middle-aged women: Results from the Study of Women's Health Across the Nation (SWAN). Menopause 2005;12:385–398. 16037753 10.1097/01.GME.0000151656.92317.A9

[9] Sierra B, Hidalgo LA, Chedraui PA. Measuring climacteric symptoms in an Ecuadorian population with the Greene Climacteric Scale. Maturitas 2005;51:236–245. 15978967 10.1016/j.maturitas.2004.08.003

[10] Garrido-Latorre F, Lazcano-Ponce EC, López-Carillo L, Hernández-Avila M. Age of natural menopause among women in México City. Int J Gynaecol Obstet 1996;53:159–166. 8735297 10.1016/0020-7292(96)02655-0

[11] Green R, Polotsky AJ, Wildman RP, et al. Menopausal symptoms within a Hispanic cohort: SWAN, the Study of Women's Health Across the Nation. Climacteric 2010;13:376–384. 20136411 10.3109/13697130903528272 PMC3268678

[12] Im EO, Lee B, Chee W, Brown A, Dormire S. Menopausal symptoms among four major groups in the US. West J Nurs Res 2010;32:540–565. 20685910 10.1177/0193945909354343 PMC3033753

[13] Malacara JM, Canto-de-Cetina T, Bassol S, et al. Symptoms at pre- and postmenopause in rural and urban women from three states of Mexico. Maturitas 2002;43:11–19. 12270577 10.1016/s0378-5122(02)00077-4

[14] Sievert LL, Huicochea-Gómez L, Cahuich-Campos D, Brown DE. Hot flashes associated with menopause in the state of Campeche, Mexico: Self-reported experience and biometric measurement. Curr Anthropol 2019;60:436–443.

[15] Schnatz PF, Kum-Whitehurst S, O'Sullivan DM. Sexual dysfunction, depression, and anxiety among patients of an inner-city menopause clinic. J Women's Health (Larchmt) 2010;19:1843–1849. 20677995 10.1089/jwh.2009.1800

[16] Thakur M, Kaur M, Kishore Sinha AK. Assessment of menopausal symptoms in different transition phases using the Greene Climacteric Scale among rural women of North India. Ann Hum Biol 2019;46:46–55. 30822155 10.1080/03014460.2019.1587508

[17] Hoehne CG, Hondula DM, Chester MV, et al. Heat exposure during outdoor activities in the US varies significantly by city, demography, and activity, Health Place 2018;54:1–10. 10.1016/j.healthplace.2018.08.014 30199773

[18] Davis RE, Hondula DM, Patel AP. Temperature observation time and type influence estimates of heat-related mortality in seven U.S. cities. Environ Health Perspect 2016;124:795–804. 26636734 10.1289/ehp.1509946 PMC4892923

[19] Kovats RS, Hajat S. Heat stress and public health: A critical review. Annu Rev Public Health 2008;29:41–55. 18031221 10.1146/annurev.publhealth.29.020907.090843

[20] DeMeyers, C, Warpinski C, Wutich A. Urban water insecurity: A case study of homelessness in Phoenix, Arizona. Environ Justice 2017;10:72–80.

[21] Olufemi O. Health of the homeless street women in South Africa. Habitat Int 1999;23:481–493.

[22] Singh GK. Area deprivation and widening inequalities in US mortality, 1969–1998. Am J Public Health 2003;93:1137–1143. 12835199 10.2105/ajph.93.7.1137 PMC1447923

[23] Vasconcelos-Raposo J, Coelho E, Fernandes HM, Rodrigues C, Maria H, Teixeira C. Factor structure and normative data of the Greene Climacteric Scale among postmenopausal Portuguese women. Maturitas 2012;72:256–262. 22578692 10.1016/j.maturitas.2012.04.003

[24] Travers C, O'Neill SM, King R, Battistutta D, Khoo SK. Greene Climacteric Scale: Norms in an Australian population in relation to age and menopausal status. Climacteric 2005;8:56–62. 15804732 10.1080/13697130400013443

[25] Chen RQ, Davis SR, Wong CM, Lam TH. Validity and cultural equivalence of the standard Greene Climacteric Scale in Hong Kong. Menopause 2010;17:630–635. 20130493 10.1097/gme.0b013e3181ca0adb

[26] Sood R, Kuhle CL, Kapoor E, et al. Association of mindfulness and stress with menopausal symptoms in midlife women. Climacteric 2019;22:377–382. 30652511 10.1080/13697137.2018.1551344

[27] Schneider A, Breitner S. Temperature effects on health—Current findings and future implications. EBioMedicine 2016;6:29–30. 27211545 10.1016/j.ebiom.2016.04.003 PMC4856774

[28] Nicolay M, Brown ML, Lalynytchev JR. A study of heat related illness preparedness in homeless veterans. Int J Disast Risk Re 2016;18:72–74.

[29] Petitti DB, Hondula DM, Yang S, Harlan SL, Chowell G. Multiple trigger points for quantifying heat-health impacts: New evidence from a hot climate. Environ Health Perspect 2016;124:176–183. 26219102 10.1289/ehp.1409119 PMC4749077

[30] Putnam H, Hondula DM, Urban A, Berisha V, Iñiguez P, Roach M. 2018. It's not the heat, it's the vulnerability: Attribution of the 2016 spike in heat-associated deaths in Maricopa County, Arizona. Environ Res Lett 2018;13:094022.

[31] Rhee J, Fabian MP, Ettinger de Cuba S, et al. Effects of maternal homelessness, supplemental nutrition programs, and prenatal PM2.5 on birthweight. Int J Environ Res Public Health 2019;16:4154. 31661898 10.3390/ijerph16214154 PMC6862522

[32] Moorkath F, Vranda MN, Naveenkumar C. Women with mental illness—An overview of sociocultural factors influencing family rejection and subsequent institutionalization in India. Indian J Psychol Med 2019;41:306–310. 31391661 10.4103/IJPSYM.IJPSYM_123_19 PMC6657485

[33] Ahmed RA, Angel C, Martell R, Pyne D, Keenan L. The impact of homelessness and incarceration on women's health. J Correct Health Care 2016;22:62–74. 26672120 10.1177/1078345815618884

[34] Fazel S, Khosla V, Doll H, Geddes J. The prevalence of mental disorders among the homeless in western countries: Systematic review and meta-regression analysis. PLoS Med 2008;5:e225. 19053169 10.1371/journal.pmed.0050225 PMC2592351

[35] Dennerstein L, Astbury J, Morse C. World Health Organization. Division of Family Health & World Health Organization. Division of Mental Health. Psychosocial and mental health aspects of women's health/Lorraine Dennerstein, Jill Astbury, Carol Morse. WHO. 1993. Available at: https://apps.who.int/iris/handle/10665/61376 Accessed January 15, 2021.

